# The impact of the 2022 spring COVID-19 booster vaccination programme on hospital occupancy in England: An interrupted time series analysis

**DOI:** 10.1371/journal.pgph.0002046

**Published:** 2024-03-06

**Authors:** Vageesh Jain, Gabriele Kerr, Thomas Beaney

**Affiliations:** 1 Royal Free London NHS Foundation Trust, London, United Kingdom; 2 Department of Primary Care and Public Health, Imperial College London, London, United Kingdom; City University of Hong Kong Jockey Club College of Veterinary Medicine and Life Sciences, HONG KONG

## Abstract

Regular booster vaccination programmes help protect the most vulnerable from COVID-19 and limit pressure on health systems. Existing studies find booster doses to be effective in preventing hospital admissions and deaths but focus on individual effects, failing to consider the population impact of incomplete vaccination coverage and seasonal patterns in disease transmission. We estimated the effectiveness of the 2022 spring booster vaccination programme, available for those aged 75 years and older, residents in care homes, and adults with weakened immune systems, on COVID-19 hospital bed occupancy in England. Booster vaccine coverage in the eligible population increased rapidly in the months after rollout (from 21^st^ March 2022), flattening out just below 80% by July 2022. We used interrupted time series analysis to estimate a 23.7% overall reduction in the rate of hospital occupancy for COVID-19 following the programme, with a statistically significant benefit in the 6–12 weeks following rollout. In the absence of the programme, we calculate that a total of 380,104 additional hospital bed-days would have been occupied by patients with COVID-19 from 4^th^ April to 31^st^ August 2022 (95% CI: –122,842 to 1,034,590). The programme delayed and shortened the duration of the peak while not reducing its magnitude. In sensitivity analyses adjusting the start of the post-intervention period or removing the rate of COVID-19 infection in the over 60s from the model, the effect of the spring booster programme on hospital bed occupancy remained similar. Our findings suggest that timing is a critical consideration in the implementation of COVID-19 booster programmes and that policymakers cannot rely on intermittent booster vaccination of high-risk groups alone to mitigate anticipated peaks in hospital pressure due to COVID-19 epidemics.

## Introduction

Protection conferred by mRNA vaccines against moderate and severe COVID-19 wanes by four to five months after a third (or first booster) dose [1]. For this reason, a ‘spring booster’ programme was implemented in the United Kingdom (UK) from late March 2022, to provide an additional booster vaccine to the most vulnerable. This was offered to those aged 75 years and older, residents in care homes for older people, and those with weakened immune systems, most of whom received their first booster dose at least six months prior. The programme was quickly rolled out, with more than 1.3 million vaccinated in under two months [2].

A 2022 case-control study from the United States (US) compared 45,903 people admitted to hospital with COVID-19 to over 200,000 controls with COVID-like illness who tested negative [1]. A fourth dose (i.e., a second booster) improved vaccine effectiveness against hospital admission by 72% in the 50–64-year age group and 76% in the 65 years and older group. Among immunocompromised individuals, vaccine effectiveness was lower (48%), and waning was more noticeable. A 2022 Canadian study used a similar design with 13,654 residents of long-term care facilities aged 60 or over [3]. They found those who had a fourth dose had a 40% lower risk of hospital admission or death, compared to those who had received only a third dose, 84 or more days prior.

The size and definitions of population groups most vulnerable to COVID -19 vary across countries [4]. In the UK, around 5.5 million people were eligible for a fourth dose by the end of June 2022 [5]. Unlike the original Joint Committee on Vaccination and Immunisation (JCVI) priority groups for COVID-19 vaccination, this did not include frontline health and social care workers [6]. JCVI advised that a spring COVID-19 booster vaccination programme would help to mitigate pressure on hospitals, despite plans for a further Autumn booster programme to protect against an anticipated Winter surge [7]. COVID-19 cases in the UK remained relatively low over the summer period, peaking at a 7-day average count of 25,000 in mid-July [8]. Although evidence supports the regular use of boosters due to waning immunity, it remains unclear exactly when and to whom booster doses should be offered through a national COVID-19 vaccination programme.

According to the November 2022 UK Health Security Agency (UKHSA) COVID-19 vaccine surveillance report, “post implementation real world vaccine effectiveness studies are needed to understand vaccine effectiveness against different outcomes” [9]. In this study, we use a quasi-experimental method to understand the impact of the spring booster programme on hospital bed occupancy rates, accounting for pre-intervention trends and confounders. Our study provides evidence on the real-world impact of the programme to help guide policy and planning on future national COVID-19 booster programmes.

## Methods

### Study design

Our interrupted time series approach used hospital bed occupancy rates for COVID-19 over time as the outcome variable. This was defined as the number of hospital beds occupied by patients with confirmed COVID-19 in England. This included patients admitted with a recent positive polymerase chain reaction (PCR) or lateral flow device test (i.e. in the last 5 days) as well as those who were admitted for reasons other than COVID-19 but had a positive test in hospital. We used this time series to model the underlying trend and construct a hypothetical scenario under which the spring booster programme had not taken place, with the underlying trend continuing unchanged to produce a counterfactual. This counterfactual was compared to observed hospital occupancy rates after the introduction of the spring booster programme, allowing us to evaluate the impact of the programme.

Our study makes two key assumptions. First, we assume that there are no other concurrent interventions or policies that would substantially affect COVID-19 hospital occupancy rates in the post-intervention period, other than the spring booster programme. This means that in the absence of the booster programme, we assume the pre-intervention trend would have continued unaltered to construct our counterfactual. Second, we assume that there are no unobserved time-varying confounders (e.g., significant changes in population characteristics), that could affect our model. Given the short study period and the strong theoretical link between vaccination rates and hospital occupancy, we believe these to be reasonable assumptions.

### Data sources

A longitudinal dataset was constructed using national-level data on the total number of hospital beds occupied by patients with confirmed COVID-19 in England, from 1^st^ August 2021 to 31^st^ August 2022 [10]. We used 4^th^ April 2022 as the cut-off date between pre- and post-intervention period, two weeks after the spring booster programme began, given evidence suggests vaccine effectiveness peaks around 2–4 weeks from administration [11]. We obtained data on population coverage of first, second, and third dose vaccination (i.e., prior to the spring booster programme) as well as daily COVID-19 rolling average case rates and genetic sequencing data on COVID-19 variants, from the open-access UK government COVID-19 database [8].

### Statistical analysis

We used a Multivariate Seasonal Autoregressive Moving Average (MSARMA) model to evaluate the impact of the COVID-19 booster vaccination programme on hospital bed occupancy. Our primary outcome was the daily rates of hospital bed occupancy. Two periods of interest were defined: 1) the pre-intervention period: from 01/08/2021 to 01/04/2022, and 2) the post-intervention period, from 02/04/2022 to 27/08/2022. We calculated the overall impact of the programme by comparing a forecasted counterfactual based on the pre-intervention trend, and assuming the absence of a booster programme. The above model was used to forecast the expected number of hospital beds in the post-intervention period assuming no intervention had occurred. The difference between the counterfactual forecast and observed hospital occupancy provided us with estimates of the additional number of hospital beds available due to the booster programme.

In our main model, we modelled pre-intervention data with the forecast R package [12], based on an approach used to model hospital bed occupancy by Eyles et al [13]. MSARMA models were used to allow for both weekly and annual seasonality in the data. An Inspection of auto-correlation function plots of hospital bed occupancy in the pre-intervention period revealed significant autocorrelation over time, and partial auto-correlation function plots revealed there was no significant direct autocorrelation between current values and values from two or more days prior (S1 Fig). Augmented Dickey Fuller and Kwiatkowski–Phillips–Schmidt–Shin tests showed the time series data was stationary. The ‘auto.arima’ function from the forecast R package (in R version 4.3.0 [14]) was applied to the log-transformed data series and used to select the best model, based on the lowest Akaike Information Criterion. The final model used an autoregressive order (p) of 2 and a moving average (q) order of 1. Co-variates included in the model were the day of the week, the proportion of the population with a first dose of COVID-19 vaccination, lagged by 14 days, and the rate of COVID-19 infection in the English population aged 60 and over. Further details of model diagnostics can be found in S2 Fig.

## Results

**Fig 1:**
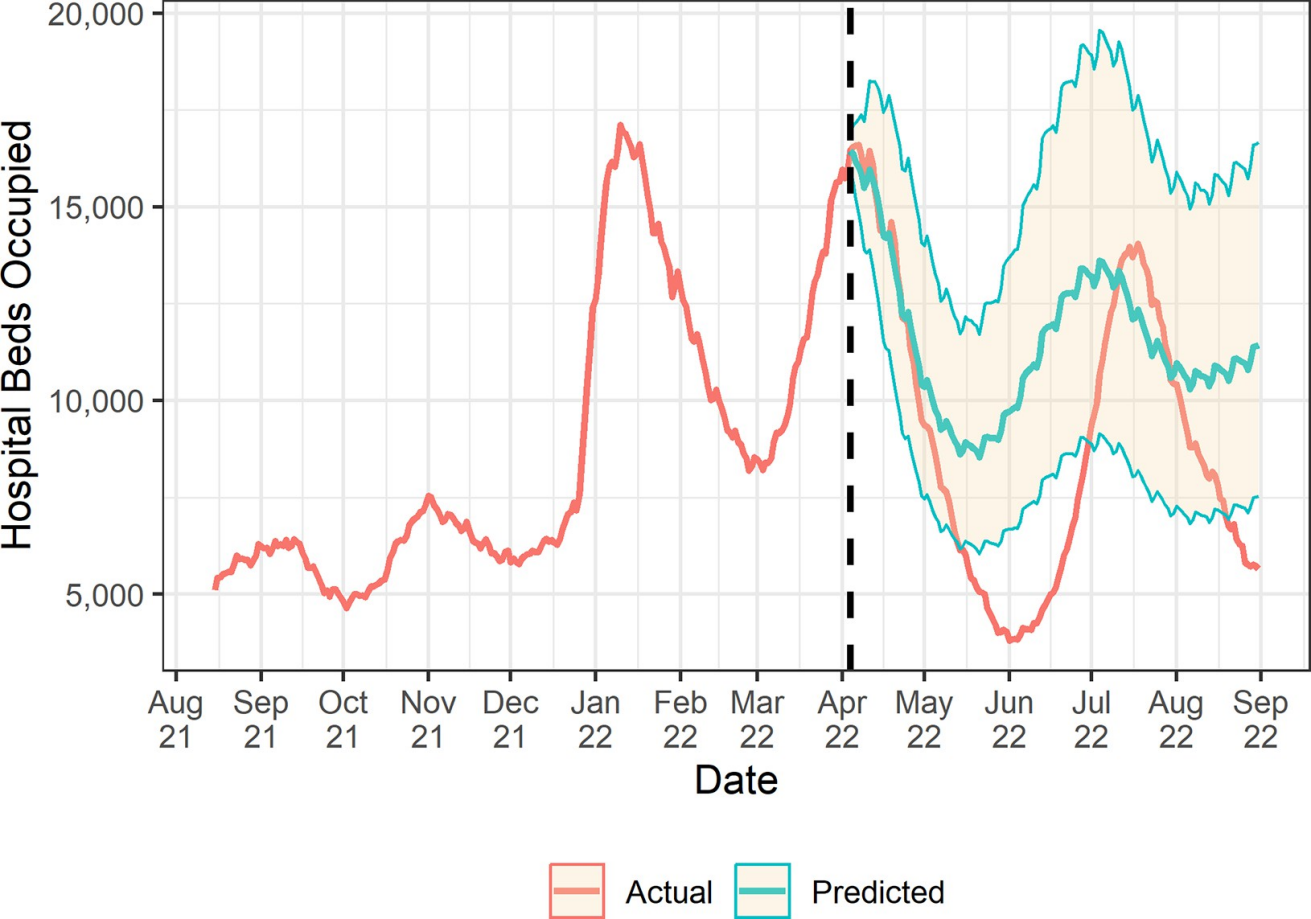
The impact of the spring COVID-19 booster programme on hospital bed occupancy in England. The pre- and post- intervention periods are delineated by a dashed line at 4^th^ April 2022. Shaded regions indicated 95% confidence intervals of model forecasts.

In the 13 months from 1^st^ August 2021 to 31^st^ August 2022, a total of 3,442,509 COVID-19 hospital bed-days were observed (95% CI 3,299,011 to 3,586,007), with two small initial peaks followed by three larger ones (Fig 1). Total daily occupancy was a median of 7,135 beds (IQR 5,972 to 11,500), rising significantly from below 8,000 per day in late 2021 to 17,120 in January 2022 and 16,600 in April 2022 (S4 Fig). Spring booster coverage increased rapidly from 0.2% on the first day of the programme (21^st^ March) to 6.5% by the end of week one and 15.7% by the end of week two. There was a rapid increase followed by a flattening out at just under 80% coverage by July 2022 (S5 Fig). There was no missing data in the daily hospitalisation or exposure variables. Third dose vaccine coverage increased quickly initially, reaching 60% between 1^st^ October 2021 and 4^th^ Jan 2022, then plateauing to a maximum of 69.2% by 31^st^ August 2022 (S6 Fig). COVID-19 case rates in those aged 60 and older varied over time, with three distinct peaks in January 2022, April 2022, and July 2022, with each successive peak being smaller than the last (S7 Fig).

**Fig 2:**
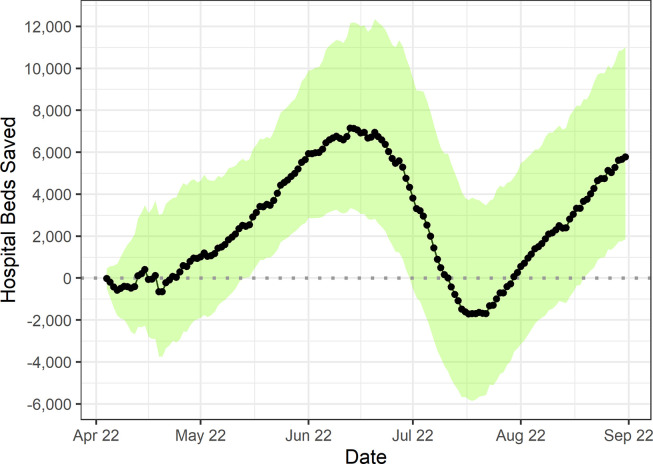
Average number of daily occupied hospital beds saved, per day. Shaded regions indicate 95% Confidence intervals of model forecasts.

Our primary model found a 23.7% (95% CI: -6.0–62.3%) overall reduction in the rate of hospital occupancy for COVID-19 in the 22 weeks following the spring booster programme. Fig 1 shows the difference between the observed rate of hospital occupancy, and that predicted by our model in the post-intervention period. The booster vaccination programme was estimated to have delayed the July 2022 peak in hospital bed occupancy and shortened its duration, although the magnitude of the peak remained similar. In the absence of the spring booster programme, we calculate that a total of 380,104 additional hospital bed-days would have been occupied by patients with COVID-19 from 4^th^ April to 31^st^ August 2022 (95% CI -122,842 to 1,034,590). Fig 2 shows that this benefit varied over time, with estimates of hospital bed days saved being statistically significant and greatest in magnitude in the 6–12 weeks following programme rollout. Model specifications are given in S1 Table.

Sensitivity analysis found that the effect of the spring booster programme on hospital bed occupancy remained robust after altering model specifications (S2 Table). If instead the post intervention period began four weeks after the start of the intervention, the predicted effect of the intervention remained similar (S3 Fig), with a predicted reduction of 25.9% (95% CI –3.1 to 63.4%) for the period 18th April to 31st August 2022, compared to an estimate of 26.2% (95% CI –5.3 to 67.5%) from the primary model. Removing the rate of COVID-19 infection in the over 60s as a predictor from the model resulted in a similar estimated reduction to the primary model but produced an overall poorer model fit (S2 Table).

## Discussion

### Key findings

Our results suggest that the spring booster vaccination programme in England led to a modest fall in hospital bed occupancy by patients with COVID-19, with a statistically significant benefit seen in the 6–12 weeks following programme rollout. The peak in hospital bed occupancy was delayed and shortened, despite reaching a similar magnitude. Approximately 380,104 fewer beds were predicted to have been occupied over the four months after the programme started. This equates to an average of 2,534 beds per day from programme start to the end of August, 2.5% of the total estimated 103,057 general and acute hospital beds available in England at the time [15]. Controlling for COVID-19 case rates in those aged 60 and above did not attenuate the effect of the spring booster programme, suggesting that boosters reduced risk of severe disease rather than risk of infection alone.

### Comparison with existing literature

The decrease in hospital bed occupancy found in our study is less than might be expected from reported estimates of fourth dose vaccine effectiveness. The UK Health Security Agency estimates vaccine effectiveness against hospitalisation with the Omicron variant to peak at 59% 2–4 weeks after a fourth dose and fall to 11% at 20 weeks [16]. A previous test negative case-control study found that effectiveness was 76% within two months after a fourth dose but waned to 70% by four months, with larger differences for a third dose [1]. Our real-world data suggest that the impact of the vaccination programme at the population level depends not only on waning levels of immunity. but upon temporal variation in COVID-19 epidemics, related to seasonality and social dynamics. Furthermore, estimates of fourth dose effectiveness are relative to people who received three doses, who may have some residual protection from earlier vaccines. Although most of those eligible for the Spring booster would likely have had a previous booster dose in Winter 2021, peak coverage for the third dose was approximately 70%. Real-world levels of immunity in the population eligible for the fourth dose were therefore lower than populations studied in test negative case-control studies.

Population coverage of the fourth dose booster was also less than complete, peaking at almost 80%. Approximately one million of those eligible remained without a booster and would be at high risk as either unvaccinated or with waning immunity following an earlier vaccine. This incomplete coverage may help to explain a relatively modest impact of the booster programme on hospital occupancy rates. In addition, the high-risk group is heterogeneous. After adjusting for age, sex and other co-morbidities, the hospitalization rate for COVID-19 in those with dementia is almost three times as high as in those without [17]. There is evidence that vaccine uptake rates can be lower in the very elderly, despite their clinical risk [18, 19]. This can be due to concerns about side effects or not being well enough to receive a vaccine. Although individual data on fourth dose vaccine recipients were not available, if the group who did not receive the Spring booster dose included a disproportionate number very high-risk individuals this may help to explain the relatively modest impact of the booster programme. It is likely that the very-high risk would also have a longer length of stay, reducing the impact of vaccines on hospital occupancy rates.

We included all patients admitted to hospital with a positive COVID-19 test, as well as incidental cases, which may provide lower estimates of vaccination programme impact. A 2022 test-negative case-control study in England identified that using a stricter definition of a COVID-19 hospitalisation with at least a two day stay in hospital and a primary diagnosis of a respiratory disease resulted in higher estimates of third dose vaccine effectiveness, compared with including all COVID-19 cases [20]. This is likely due to a reduction in the number of incidental cases included, which may be higher for the Omicron variant compared to previous variants. It is likely that estimates of COVID-19 bed occupancy in our study would be lower using this stricter definition, but data on primary diagnosis were not available, preventing comparison.

### Strengths and limitations

Interrupted time series (ITS) designs are robust quasi-experimental designs commonly used to evaluate the impact of public health interventions and programmes [21]. To our knowledge, this is the first real-world evaluation of the spring booster vaccination programme in England, directly answering a call from experts at UK Health Security Agency to make use of routine data to understand the impact of COVID-19 vaccination programmes [9]. We go beyond individuals or groups to look at impact across the national population, which accounts for both individual-level vaccine effectiveness, but also population coverage and temporal variation in disease transmission, critical in providing the type of evidence required to inform future public health policy.

Our study has some important limitations. First, although we use over a year of daily data on hospital bed occupancy, the time series method could be improved with additional data to strengthen the validity of the counterfactual prediction. Nevertheless, further extension of the time period to when the Omicron variant was not dominant may not improve the model. Secondly, there may be additional factors which affect COVID-19 hospital bed occupancy variably over time, which we have been unable to account for in our analysis and which might lead to under- or over-estimation of the effect of the programme. This includes hospital pressures and changes to admission thresholds, which may impact on hospital occupancy rates independent of the severity of disease in individuals [22]. Although we did not explicitly control for this, our analysis excludes Winter 2020/21, which was the key period where the English hospital system was under great pressure. The availability of COVID-19 treatments varied over time and may also have affected rates of hospital occupancy. However, most available treatments used for COVID-19 are used in already hospitalized patients to reduce severity and the need for ICU admission. Of the drugs available in the community in the UK to prevent hospital admission, all were available prior to our study period [23]. Finally, we only investigated one outcome measure (hospital bed occupancy), but there are several other measures of the impact of COVID-19 vaccination programmes, including hospitalization, intensive care admissions, COVID-19 mortality, and morbidity, including long COVID. Such evidence must be considered alongside our study.

### Implications for policy and practice

The vaccination programme appeared to delay and shorten the peak in hospital bed occupancy and prevented most hospital bed-days in the 6–12 weeks following the rollout. This suggests that the timing of COVID-19 booster programme rollouts is an important consideration for future epidemic preparedness. Nevertheless, the programme did not have a large impact on the magnitude of the peak, suggesting that policymakers cannot rely on intermittent booster vaccination of high-risk groups alone to mitigate anticipated peaks in hospital pressure. Improved vaccine efficacy and coverage could increase impact, but additional public health interventions to protect high-risk groups and control community transmission will be needed to limit avoidable hospital bed occupancy.

In May 2022, the UK government stated that eventually they wanted to “manage COVID-19 like other respiratory illnesses, while minimising mortality and retaining the ability to respond if a new variant emerges with more dangerous properties than the Omicron variant, or during periods of waning immunity.” [24] If steady levels of community transmission of COVID-19 and predictable seasonal peaks remain, intermittent vaccination will be necessary. As for influenza, a regular vaccine will need to be offered to high-risk populations to limit preventable infections, hospital admissions, and deaths. Existing real-world evaluations of COVID-19 vaccination programmes measure performance against uptake and the process of vaccine delivery [25]. Our findings allow the expansion of key performance indicators to set realistic expectations of the impact of future national booster programmes on hospital occupancy in the few months following rollout.

In countries such as China, epidemiological scenario modelling continues to be a vital tool to inform the COVID-19 response [26]. In England, epidemiological modelling has become less prominent [27] but will be critical if future novel variants arise. Most epidemiological scenario models use hospital admission rates as a core outcome measure (alongside infections and deaths), rather than occupancy rates [28]. Prominent studies investigating vaccine effectiveness also fail to investigate impact on occupancy [29]. Our estimates allow future models to expand their scope to include both COVID-19 admission and hospital occupancy rates. Since occupancy rate is a combination of admissions and length of stay, it provides valuable information on how vaccines affect the severity of illness. To guide capacity and resource deployment plans during an epidemic, decision-makers need to know not only how many patients to expect requiring assessment, but also how many beds they will have available over a longer period [30]. By assessing the impact of the Spring booster on hospital occupancy rate, we usefully build on the existing evidence base to work towards a more comprehensive understanding of the impact of COVID-19 vaccines on health systems.

## Conclusions

Existing studies investigating the effectiveness of fourth dose COVID-19 vaccination fail to capture the overall impact of real-world programmes. Population impact depends not only on effect of vaccines at an individual level, but also on vaccine coverage and seasonal trends in disease transmission, with a large minority of those eligible not receiving booster doses or only partially taking up previous vaccination offers. We found that the spring booster programme saved an estimated 380,000 COVID-19 bed-days over four months, with a statistically significant benefit seen in the 6–12 weeks following programme rollout. The peak in hospital bed occupancy following the programme rollout was delayed and shortened but was similar in magnitude to that expected in the absence of the programme. This study highlights the need to optimally time the rollout of booster programmes to ease hospital pressures, to support vaccination with other public health interventions, and the added value of using routine data to evaluate the impact of national programmes.

## Supporting information

S1 Fig Log-transformed data series of occupied hospital beds (top plot) and autocorrelation function (ACF) and partial autocorrelation function (PACF) plots (bottom plots). Autocorrelation plots show the correlation coefficient (y-axis) values in the data series have with values lagged by different intervals (x axis). Correlation coefficients outside of the blue dashed lines indicate noteworthy correlations.
(PDF)

S2 Fig Diagnostic plots for the ARMA(2,0,1) model. Residuals appear evenly dispersed around 0 with no trend (top plot) and no significant residual lag (bottom left) (Ljung-Box test: p-value 0.713 with 14 total lags used). The distribution of residuals is largely symmetrical (bottom right).
(PDF)

S3 Fig The impact of the spring COVID-19 booster programme on hospital bed occupancy in England. The pre- and post- intervention periods are delineated by a dashed line at 18^th^ April 2022. Shaded regions indicated 95% confidence intervals of model forecasts.
(PDF)

S4 Fig Density plot of hospital beds occupied by COVID-19 patients. (PDF)

S5 Fig Spring booster vaccine coverage over time in 2022. (PDF)

S6 Fig Third dose vaccine coverage over time. (PDF)

S7 Fig COVID-19 average weekly cases in 60+: Time series. (PDF)

S1 Table ARMA(2,0,1) model with errors. Predictors include coverage of the first dose of the COVID-19 vaccination and the COVID-19 case rate in the over 60s population.
(DOCX)

S2 Table ARMA(2,0,1) model with errors. Predictors include coverage of the first dose of the COVID-19 vaccination.
(DOCX)

S1 File STROBE checklist. (DOCX)
