# Supplementary figures and images for: The impact of the 2022 spring COVID-19 booster vaccination programme on hospital occupancy in England: An interrupted time series analysis
Source: PLOS Glob Public Health. 2024 Mar 6;4(3):e0002046. doi: 10.1371/journal.pgph.0002046 (PMC10917281; doi:10.1371/journal.pgph.0002046)

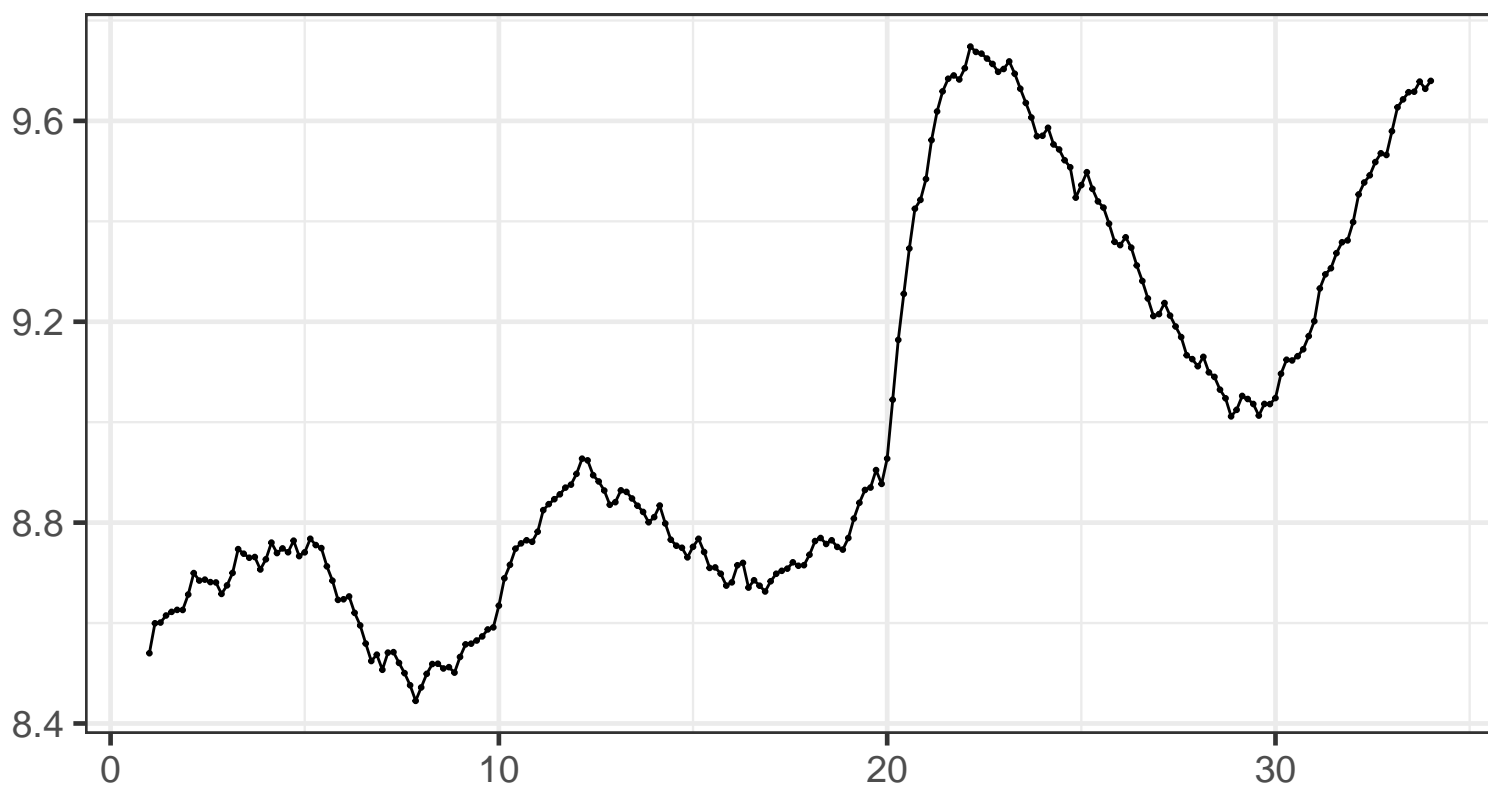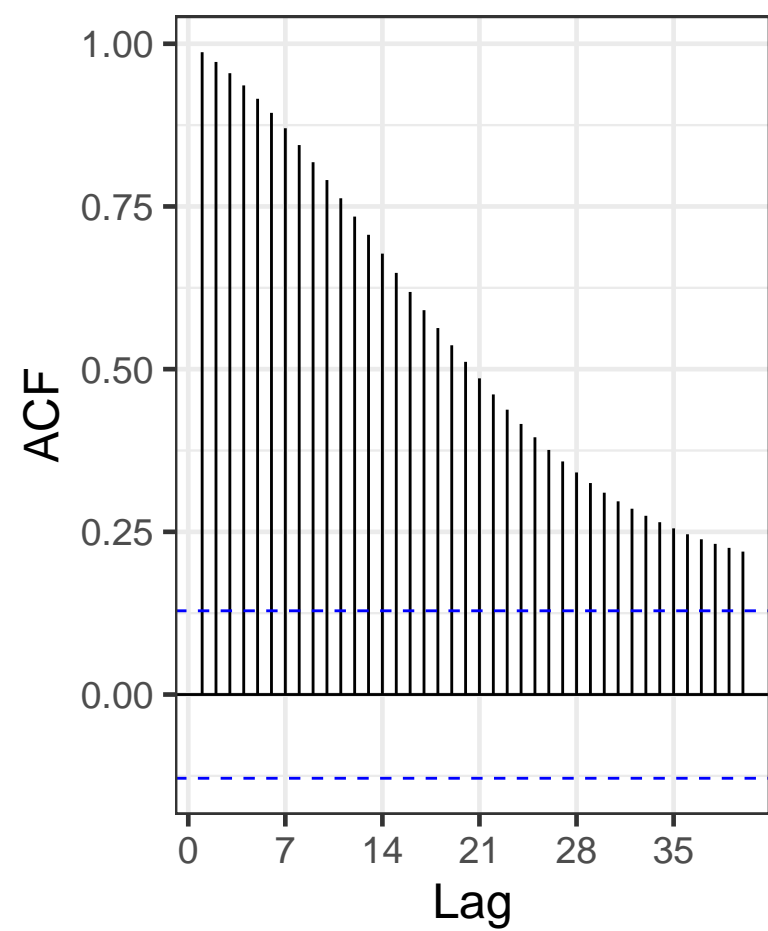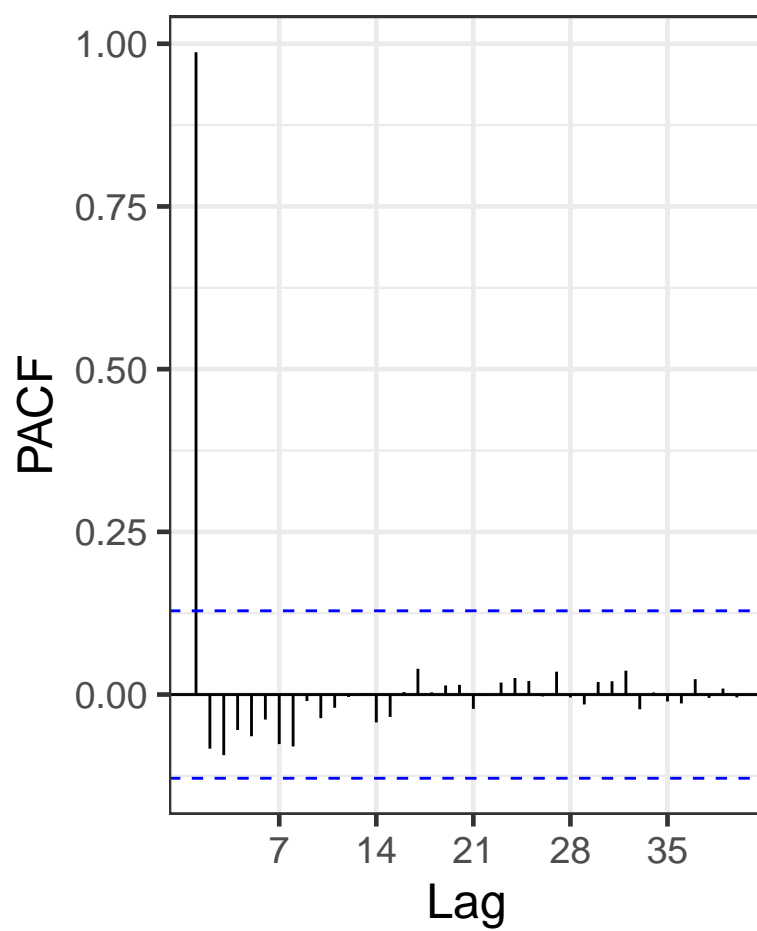

Supplement: S1 Fig — Log-transformed data series of occupied hospital beds (top plot) and autocorrelation function (ACF) and partial autocorrelation function (PACF) plots (bottom plots). Autocorrelation plots show the correlation coefficient (y-axis) values in the data series have with values lagged by different intervals (x axis). Correlation coefficients outside of the blue dashed lines indicate noteworthy correlations. (PDF) [file pgph.0002046.s001.pdf]

# Residuals from Regression with ARIMA(2,0,1) errors

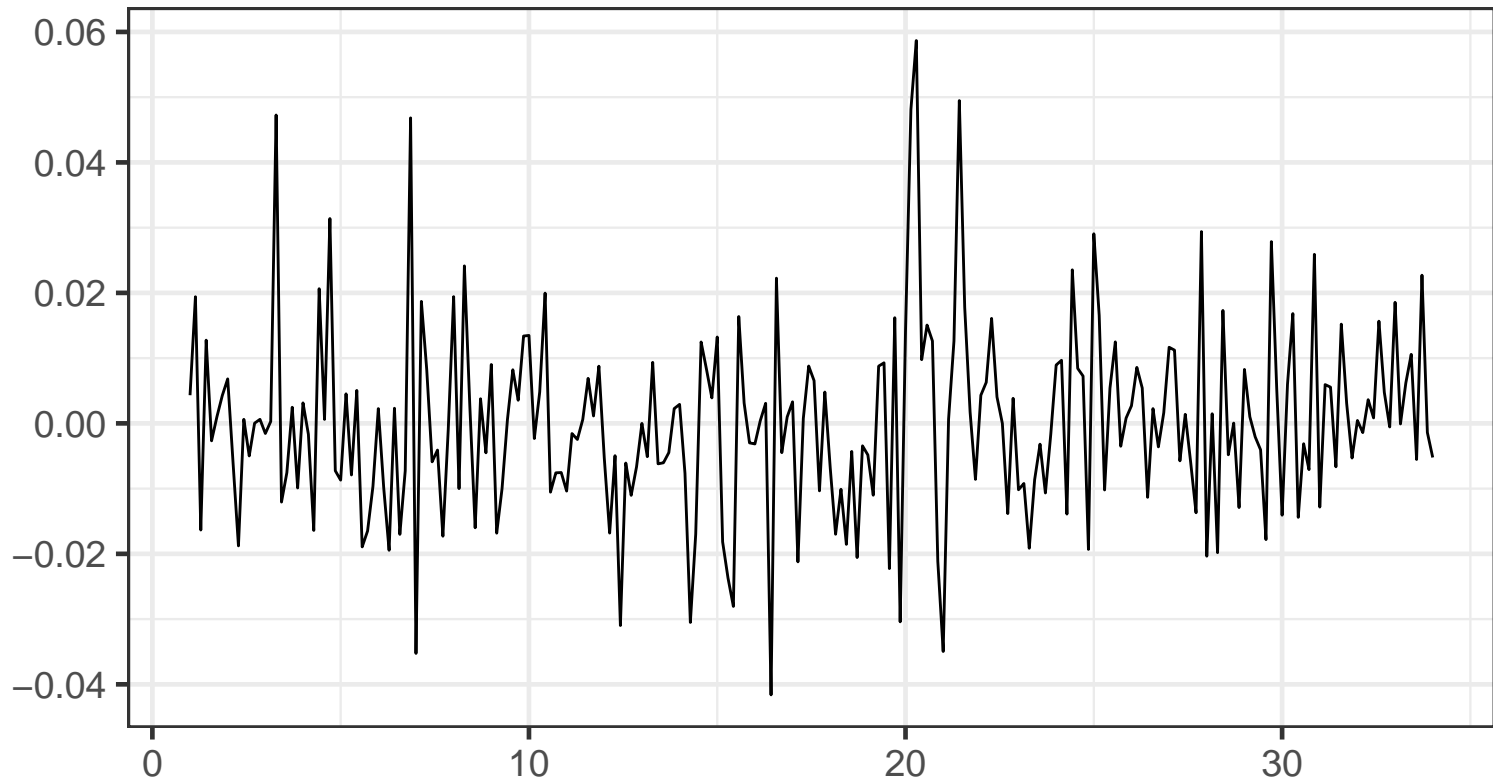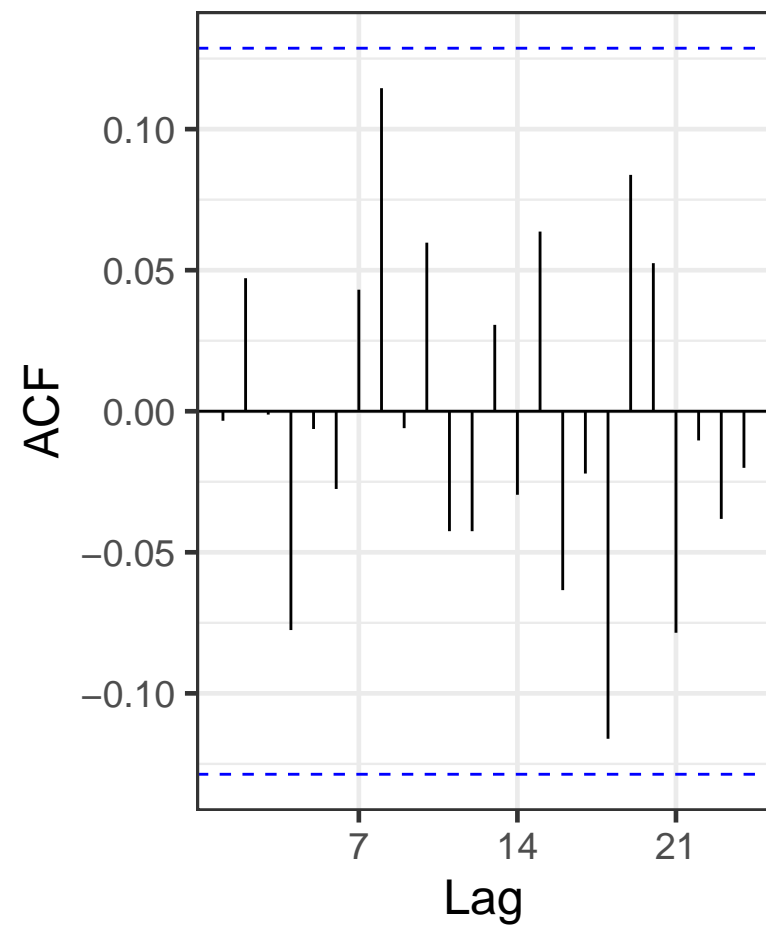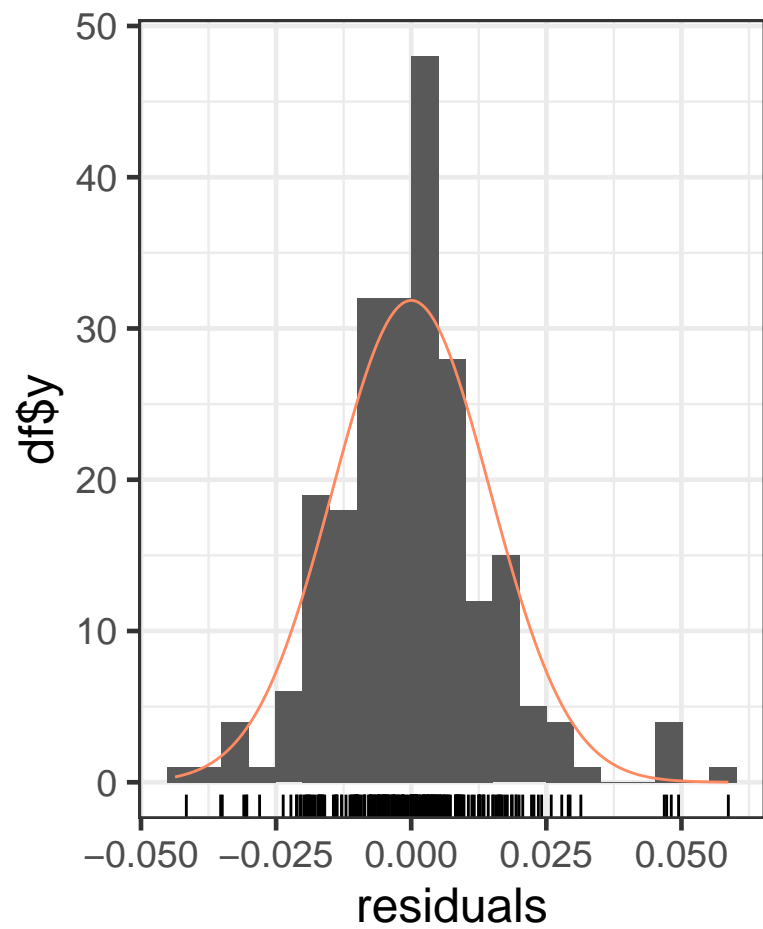

Supplement: S2 Fig — Residuals appear evenly dispersed around 0 with no trend (top plot) and no significant residual lag (bottom left) (Ljung-Box test: p-value 0.713 with 14 total lags used). The distribution of residuals is largely symmetrical (bottom right). (PDF) [file pgph.0002046.s002.pdf]

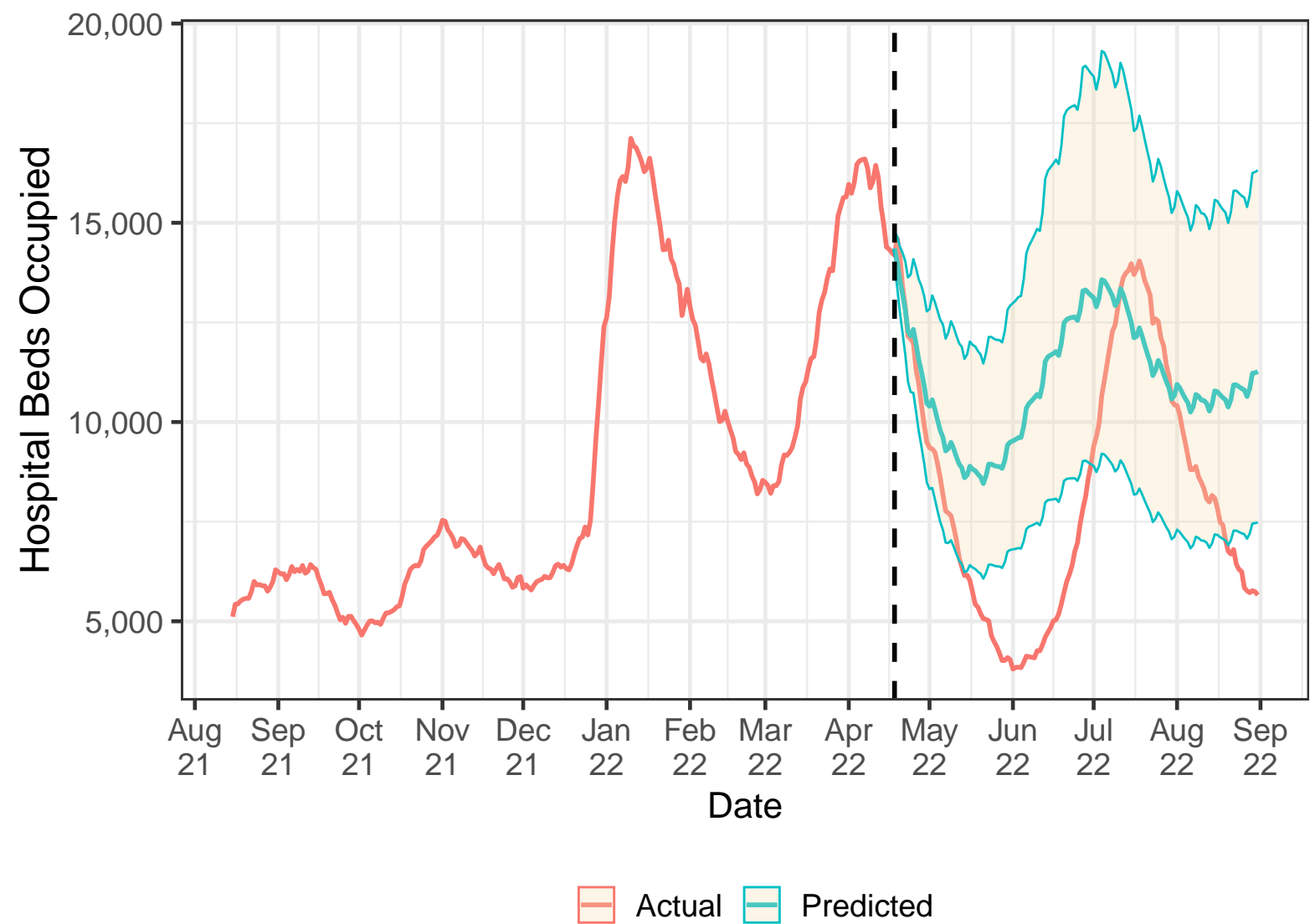

Supplement: S3 Fig — The pre- and post- intervention periods are delineated by a dashed line at 18th April 2022. Shaded regions indicated 95% confidence intervals of model forecasts. (PDF) [file pgph.0002046.s003.pdf]

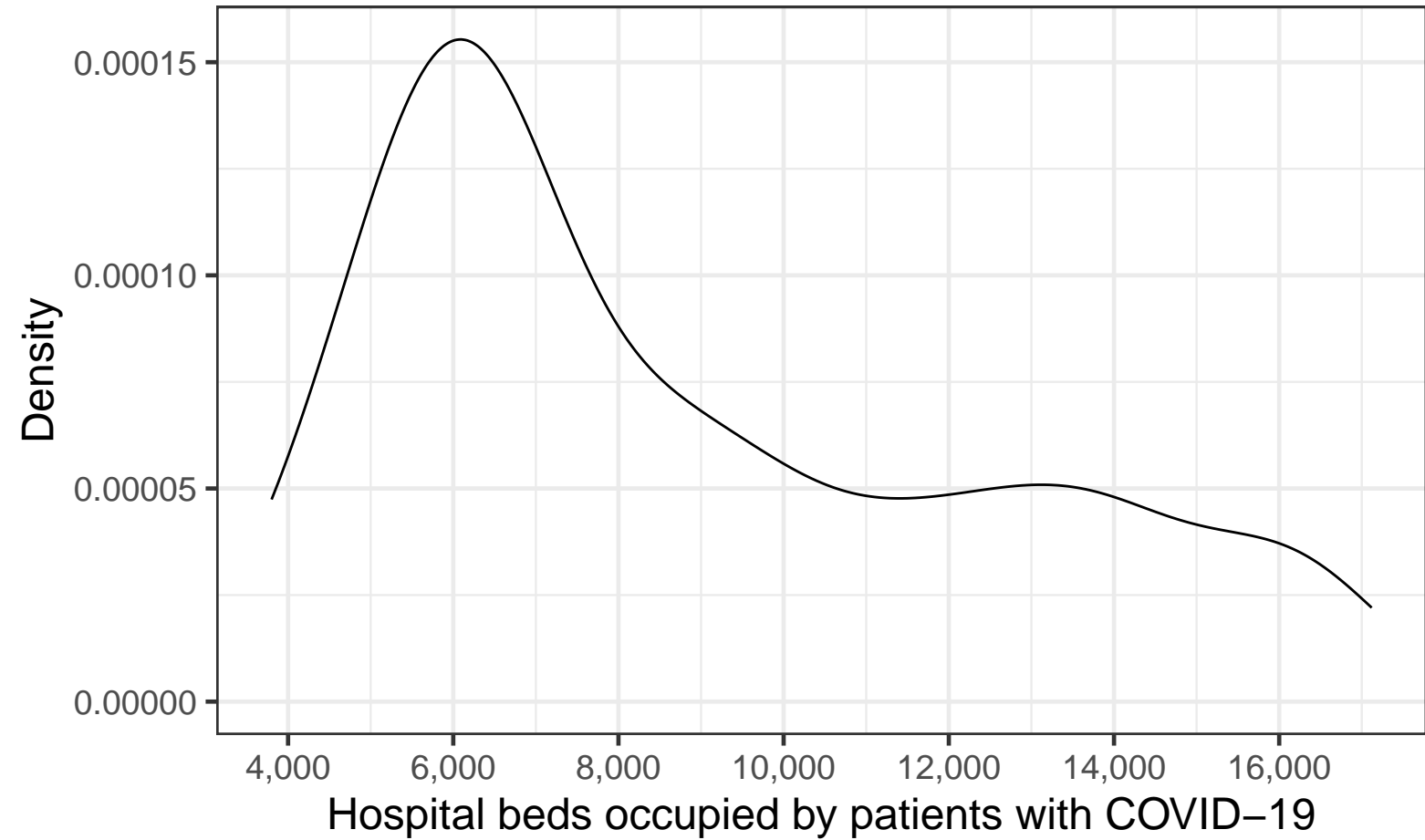

Supplement: S4 Fig — (PDF) [file pgph.0002046.s004.pdf]

Spring booster vaccine coverage  
(% eligible)

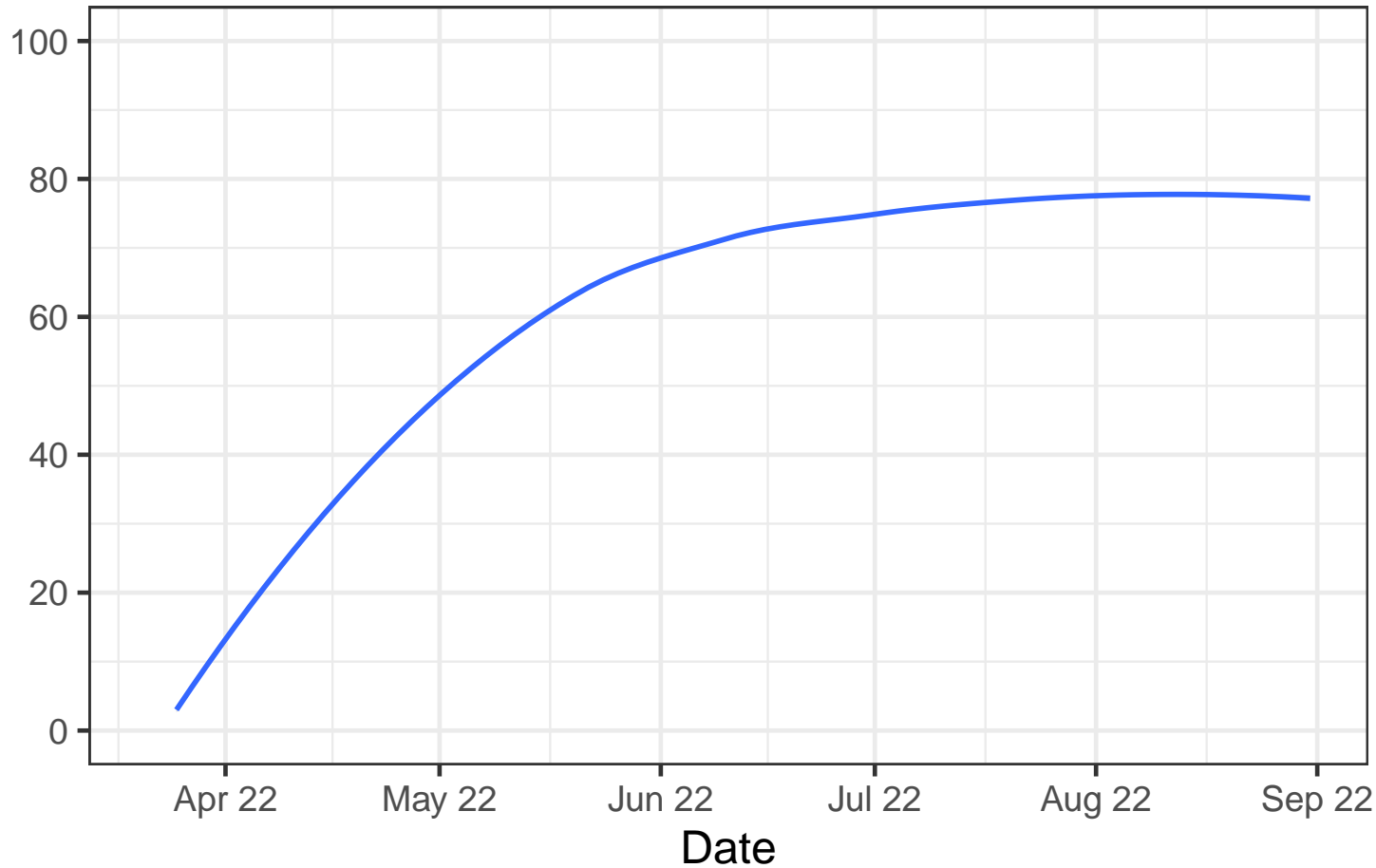

Supplement: S5 Fig — (PDF) [file pgph.0002046.s005.pdf]

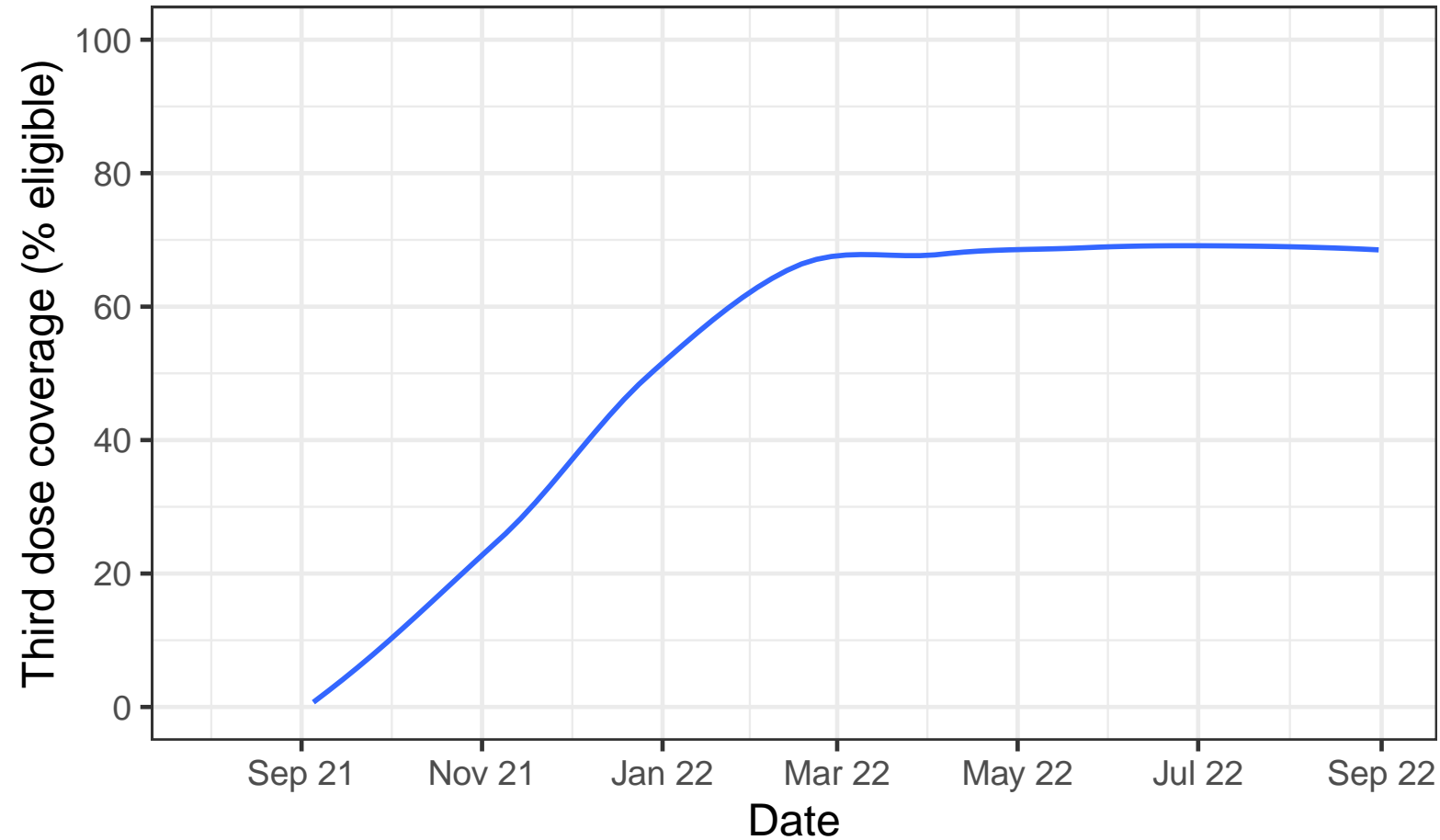

Supplement: S6 Fig — (PDF) [file pgph.0002046.s006.pdf]

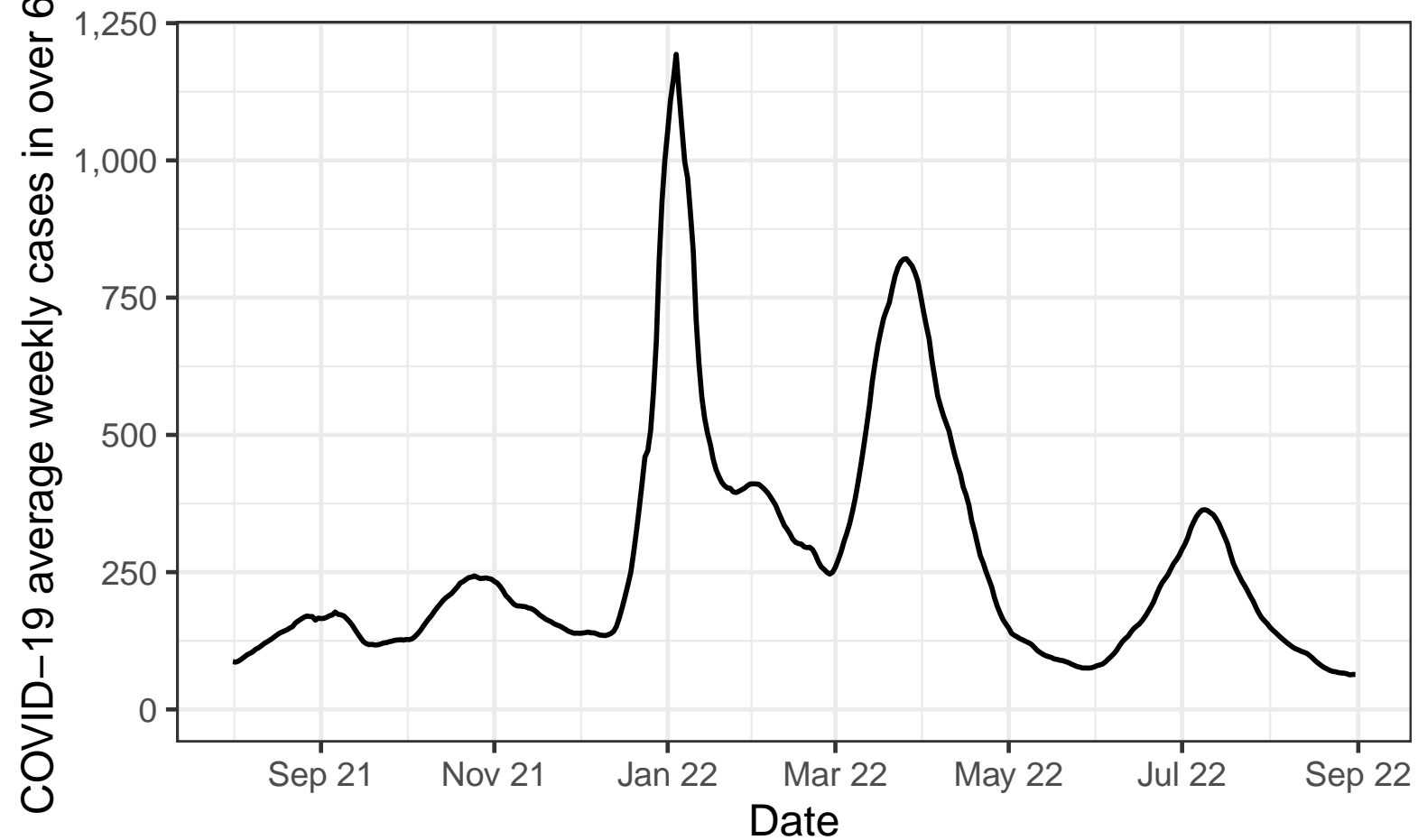

Supplement: S7 Fig — (PDF) [file pgph.0002046.s007.pdf]
