# Supplementary material for: The impact of the 2022 spring COVID-19 booster vaccination programme on hospital occupancy in England: An interrupted time series analysis
Source: PLOS Glob Public Health. 2024 Mar 6;4(3):e0002046. doi: 10.1371/journal.pgph.0002046 (PMC10917281; doi:10.1371/journal.pgph.0002046)
Supplement: S1 Table — Predictors include coverage of the first dose of the COVID-19 vaccination and the COVID-19 case rate in the over 60s population. (DOCX) [file pgph.0002046.s008.docx]

**S1 Table:** **ARMA(2,0,1) model with errors.** Predictors include coverage of the first dose of the COVID-19 vaccination and the COVID-19 case rate in the over 60s population.

|  | *Coefficient* | *Lower* | *Upper* |
| --- | --- | --- | --- |
| *ar1* | 1.968 | 1.937 | 2.000 |
| *ar2* | -0.975 | -1.006 | -0.943 |
| *ma1* | -0.766 | -0.876 | -0.656 |
| *intercept* | 2.843 | 0.856 | 4.829 |
| *Monday* | 0.031 | 0.027 | 0.035 |
| *Tuesday* | 0.028 | 0.022 | 0.033 |
| *Wednesday* | 0.020 | 0.014 | 0.026 |
| *Thursday* | 0.012 | 0.006 | 0.018 |
| *Friday* | 0.006 | 0.000 | 0.011 |
| *Saturday* | 0.015 | -0.019 | -0.011 |
| *Dose 1 coverage* | 0.069 | 0.046 | 0.092 |
| *COVID-19 rate in over 60s* | 0.000 | 0.000 | 0.001 |
| **AIC**: -1272.350  **BIC**: -1227.547  **RMSE**: 134.001 | | | |
