# Supplementary material for: The impact of the 2022 spring COVID-19 booster vaccination programme on hospital occupancy in England: An interrupted time series analysis
Source: PLOS Glob Public Health. 2024 Mar 6;4(3):e0002046. doi: 10.1371/journal.pgph.0002046 (PMC10917281; doi:10.1371/journal.pgph.0002046)
Supplement: S2 Table — Predictors include coverage of the first dose of the COVID-19 vaccination. (DOCX) [file pgph.0002046.s009.docx]

**S2 Table –** ARMA(2,0,1) model with errors. Predictors include coverage of the first dose of the COVID-19 vaccination.

|  | *Coefficient* | *Lower* | *Upper* |
| --- | --- | --- | --- |
| *ar1* | 1.952 | 1.910 | 1.995 |
| *ar2* | -0.959 | -1.002 | -0.916 |
| *ma1* | -0.637 | -0.777 | -0.497 |
| *intercept* | 2.954 | 0.027 | 5.881 |
| *Monday* | 0.031 | 0.027 | 0.036 |
| *Tuesday* | 0.028 | 0.023 | 0.034 |
| *Wednesday* | 0.021 | 0.014 | 0.027 |
| *Thursday* | 0.013 | 0.006 | 0.019 |
| *Friday* | 0.006 | 0.000 | 0.012 |
| *Saturday* | 0.015 | -0.019 | -0.010 |
| *Dose 1 coverage* | 0.069 | 0.036 | 0.102 |
| **AIC**: -1242.82  **BIC**: -1201.46  **RMSE**: 135.2973 | | | |
